# A novel method for constructing continuous intrinsic surfaces of nanoparticles

**DOI:** 10.1007/s00894-017-3378-9

**Published:** 2017-07-03

**Authors:** Daniel T. Allen, Christian D. Lorenz

**Affiliations:** Theory & Simulation of Condensed Matter Group, Department of Physics, Strand Campus, King’s College London, Strand, London WC2R 2LS UK

**Keywords:** Nanoparticles, Micelles, Molecular simulation, Interfacial properties

## Abstract

In recent years, the field of nanotechnology has become increasingly prevalent in the disciplines of science and engineering due to it’s abundance of application areas. Therefore, the ability to study and characterize these materials is more relevant than ever. Despite the wealth of simulation and modeling studies of nanoparticles reported in the literature, a rigorous description of the interface of such materials is rarely included in analyses which are pivotal to understanding interfacial behavior. We propose a novel method for constructing the continuous intrinsic surface of nanoparticles, which has been applied to a model system consisting of a sodium dodecyl sulfate micelle in the presence of testosterone propionate. We demonstrate the advantages of using our continuous intrinsic surface definition as a means to elucidate the true interfacial structure of the micelle, the interfacial properties of the hydrating water molecules, and the position of the drug (testosterone propionate) within the micelle. Additionally, we discuss the implications of this algorithm for future work in the simulation of nanoparticles.

**Graphical Abstract:**
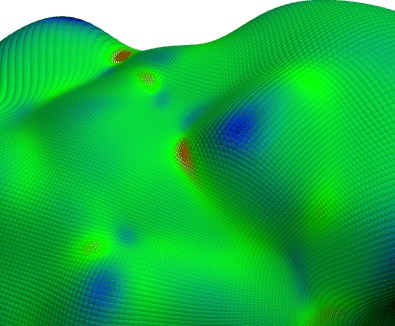
ᅟ

ᅟ

## Introduction

Nanotechnology is an emerging field of science that focuses on the synthesis and application of a variety of different materials, typically on length scales of 1-100 nm. These range from metallic nanomaterials, e.g., gold, silver, titanium zinc, copper, silver, gold, and titanium, to soft nanomaterials including polymers and surfactants. In recent years, carbon nanotubes [1], gold nanoparticles (GNPs) [2], and quantum dots [3, 4] have received a great deal of attention in the literature for their wide range of application areas including chemical manufacturing [5], energy conversion and storage [6], environmental technology [7], and biological applications [8, 9]. Biodegradable polymeric nanoparticles are also being investigated for use in biological applications including as drug delivery vehicles due to their solubilization ability, controlled release, and minimal toxicity [10, 11]. This practice of utilizing nanotechnology for medical applications has instilled tremendous confidence in the future of such techniques for improving the efficiency of diagnosis and treatment of human diseases, particularly for various forms of cancer [12, 13].

The physical and chemical properties of nanoparticles such as size, surface chemistry, surface charge, geometry/shape, hydrophobicity, and roughness can affect their resulting behavior and their effectiveness in the various applications. Therefore, the ability to characterize these attributes is of vital importance in the rational design of functional nanomaterials. Despite the vast contribution of experimental work conducted in the field, such techniques are not without their limitations, which arise predominately as a result of the small length scales of nanoparticles. To combat this, computational modeling has proven to be an invaluable tool for studying these nanoscale systems in recent years. With the relentless advancement in computational resources available on the commercial market, molecular dynamics (MD) simulations allow the study of molecular systems on increasingly large spatial and temporal scales with atomic resolution. These simulation techniques permit the study of the dynamic properties of nanoscale materials as a function of time and are able to identify molecular interactions, which are pivotal to the observed function of the system, information which is unattainable from the majority of experimental methods.

The ability to characterize and define nanoparticle interfaces in detail is highly relevant to understanding their function, as interfacial effects govern numerous important properties of nanoparticles such as hydration [14], encapsulation [15], and self-assembly behavior [16] to name but a few. Nevertheless, an accurate description of the nanoparticle interface is seldom incorporated into analysis. In this study, we propose a novel method of constructing the continuous intrinsic surface of nanoparticles and apply this to a sodium dodecyl sulfate (SDS) micelle in the presence of testosterone propionate (TP), a system which we have studied in detail in previous work [17].

SDS is a commonly studied surfactant molecule which self assembles into a variety of different aggregate structures due to its amphiphilic nature. These structures, which include micelles, monolayers, bilayers, lamellar, and other cubic phases, have wide applications in areas such as pharmaceuticals, mineral separation processes, environmental remediation, the food industry, personal care products, and petroleum recovery [18–25]. Since the first MD study of an SDS micelle published in 1990 [26], the structural and interfacial properties of these systems have been investigated in greater detail [27–34] and recently, interest in studying the encapsulation of solutes within SDS micelles has flourished with an abundance of work being produced in this area [17, 35–40]. In all of these studies, the micelle structure is quantified, at least partially, using radial density plots. These express the average density of various different atomic species as a function of their distance away from the micelle’s center of mass. However, due to the elliptical and ever-changing micelle shape, the true interfacial behavior is difficult to elucidate from radial density plots with any great detail. This means that if properties of the micellar system are to be studied rigorously in relation to the interface, then the smearing effects arising from capillary wave fluctuations must be removed in the analysis.

Such rigor has indeed been applied to surfactant monolayers through the proposition of various different methods to construct the *intrinsic surface* at the monolayer/water interface [41–44] and arbitrary liquid interfaces [45, 46]. These approaches have led to a more insightful analysis of the monolayer properties after removing the effects of surface roughness and thus permitting study of monolayer properties with respect to the interface. To the best of our knowledge, there is currently only one MD study of a surfactant micelle in the literature in which a thorough treatment of the interface has been included. The system in this study by Chowdhary et al. was an inverse micelle in the absence of any external solutes [47]. Furthermore, their resulting surface is a discontinuous function, the negative implications of which will be discussed in the remainder of the manuscript. We propose a novel method of constructing the continuous intrinsic surface of nanoparticles and compare the results obtained from the SDS+TP micelle when analysis is performed with and without utilizing the intrinsic surface and show that use of the intrinsic surface leads to a more enlightening description of physical properties.

The remainder of the manuscript is organized as follows: “Simulation details” outlines the simulation protocol used in the study, the analysis techniques that have been employed are discussed in “Analysis” including our proposed method for constructing the micelle intrinsic surface, the results are reported in “Results” and finally in “Conclusions” we summarize our findings and outline future prospects in light of our findings.

## Simulation details

The solubilization of TP within an SDS micelle in aqueous solution has been investigated using large-scale atomistic MD simulations. The aggregation numbers of SDS and TP were found to be 76 and 13, respectively, as calculated from recent neutron scattering experiments on SDS micelles in the presence of TP at equilibrium [17]. First, a spherical micelle structure was built using the Packmol software package [48] consisting of 76 SDS monomers in vacuo. This initial system was subjected to energy minimization followed by a 240 ps simulation in the NVT ensemble in which the temperature was fixed at 300 K. Next, the micelle was placed into the center of a 126 Å × 126 Å × 126 Å simulation box and was solvated with water such that the resulting concentration of SDS was 3 g/100 ml, the same as in the neutron experiments. The solvated micelle was then subjected to another energy minimization and a further 400-ps NVT simulation with the temperature fixed at 300 K.

The coordinates of the SDS micelle and all water molecules within 5 Å of the aggregate interface were taken from the final configuration of the thermalization simulation. Then, 13 TP molecules were packed into the space surrounding the micelle and interfacial water molecules. Precautions were taken to ensure that the TP molecules were positioned within the 10-AA interaction cutoff distance of the micelle, without overlapping with the interfacial water layer. Additional water molecules were packed into the box so that the total number of water molecules was equal to that of the system before the addition of TP. An energy minimization was performed, followed by three further equilibration runs. First, a 2-ns simulation in the NPT ensemble was used to equilibrate the system pressure to 1 atm; then a 100-ps NVT simulation was performed to equilibrate the temperature to 300 K, and finally a 10-ns NPT simulation was performed. This simulation protocol allows for the volume of the system to be corrected to at least close to the appropriate value after using a slightly larger box volume to pack the molecules into the simulation box, then the temperature is equilibrated to the desired temperature of 300 K and finally the size and shape of the SDS micelle is allowed to equilibrate by allowing the box size to change in concert with the micelle.

The final configuration of the SDS+TP micelle obtained from the simulation protocol outlined above was used as the starting state for the production simulation. This was performed at atmospheric pressure and at a temperature of 300 K in the NPT ensemble, from which an 80-ns trajectory was obtained. By using NPT for the production simulation, the simulation box is able to change with the size and shape of the micelle, instead of artificially creating density gradients within the water surrounding the micelle. The LAMMPS simulation package [49] was used to perform the simulation with the CHARMM force field [50, 51] used for the description of the inter- and intra-molecular interactions of SDS and TP. Interactions involving water were described using the TIP3P water model [52], which was modified for the CHARMM forcefield [53]. The van der Waals and electrostatic interactions were cut-off at 10 Å and 12 Å respectively. Long-range electrostatic interactions were computed using the PPPM method [54]. The system temperature and pressure (in the NPT simulations) were controlled using the Nosé-Hoover thermostat [55] and barostat [56] implemented in LAMMPS [57–60]. A 2-fs timestep was used in the production simulation to ensure stable integration of Newton’s equations of motion with the velocity Verlet algorithm whilst the SHAKE algorithm [61] was used to constrain all hydrogen-containing bonds. The measurements discussed in the remaining sections of this manuscript were conducted using the last 20 ns of the 80-ns production period in which the system was deemed to be stable throughout.

## Analysis

### Intrinsic surface

**Fig. 1:**
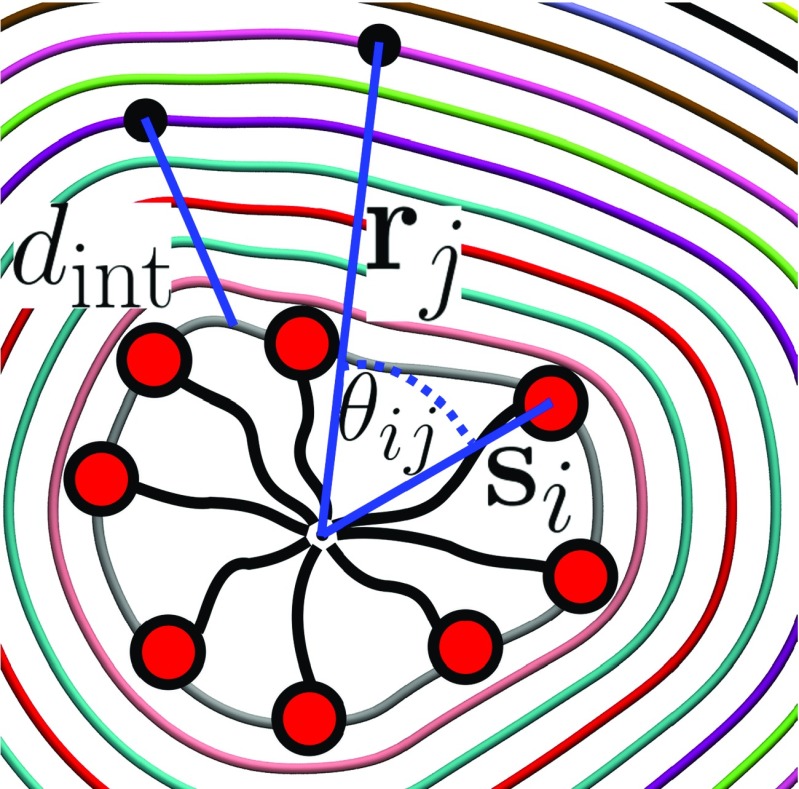
A schematic diagram showing various quantities discussed in the text: *Colored rings* represent isovalues of *d*_int_ for a particular micelle configuration; the distance *d*_int_ is shown along with the vectors **s**_*i*_, **r**_*j*_ and the angle *𝜃*_*ij*_

**Fig. 2:**
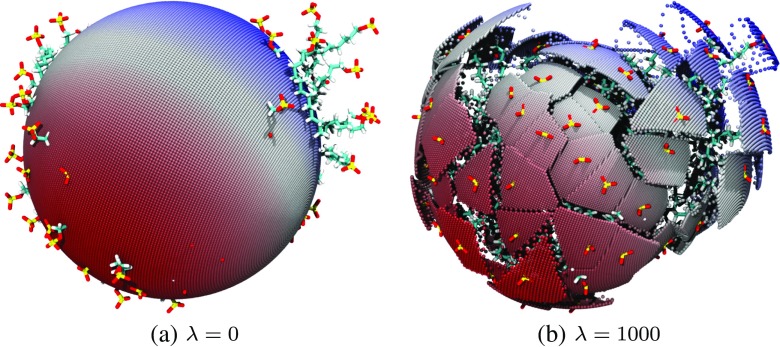
Images of the intrinsic surface constructed for the SDS micelle with *λ* values set to **a**) 0 and **b**) 1000. The micelle has the same orientation in both images, which shows the contrast in the resulting surfaces. These images highlight the dependence of the resulting surface on the choice of *λ*. Note that TP molecules have been omitted from these images for clarity

Prior to calculating the intrinsic density of a micelle, we first must establish a clear definition of the micelle/water interface. In an analogous way to many intrinsic surface constructions for surfactant monolayers [41–44], the choice of anchor points is a trivial one: the sulfur atoms in the DS ^−^ headgroups, which are the geometric center of the headgroup, play an important role in the interaction of these surfactants with their environment. When calculating the distance from an atom, *j*, to the micelle interface, let the vector pointing from the instantaneous center of mass position of the micelle, **c**_m_, to the position of *j* be denoted as **r**_*j*_. Similarly, let the vector pointing from **c**_m_ to sulfur atom *i* be denoted by **s**_*i*_. We wish to have a continuous description of the micelle intrinsic surface and so this must be well defined at any given $\vec {r_{j}}$. Moreover, we want the surface function to be smooth and continuous, absent of abrupt jumps in micelle depth like those present in [47]. To produce an intrinsic surface definition inclusive of the desired attributes stated above, the surface is calculated as a continuous function of all of the anchor points in the micelle. The magnitudes of the vectors {**s**_*i*_} provide an indication of the micelle depth at each anchor point. A weighted average is employed to determine the micelle depth at any given point, **r**_*j*_, such that the influence of anchor points is a decaying function of the angle between vectors **r**_*j*_ and **s**_*i*_, denoted by *𝜃*_*ij*_ (see Fig. 1). The distance to the intrinsic micelle surface *d*_int_ from any point **r**_*j*_ is defined mathematically as follows:

1 $$ d_{\text{int}}(\mathbf{r}_{j})=|\mathbf{r}_{j}|-\frac{\sum\limits_{i=1}^{N} \exp{[-\lambda \theta_{ij}^{2}] \cdot |\mathbf{s}_{i}| }}{\sum\limits_{i=1}^{N} \exp{[-\lambda \theta_{ij}^{2}]}} $$

where *N* is equal to the number of anchor points present in the micelle, *𝜃*_*ij*_ denotes the angle in radians between the vectors **s**_*i*_ and **r**_*j*_, and *λ* is a free parameter which determines how smooth the resulting intrinsic surface is. As *λ* → 0, the intrinsic surface tends towards a perfect sphere, the radius of which is equal to the average micelle radius, i.e., the average over the magnitudes of {**s**_*i*_}. As *λ* →*∞*, the surface tends towards that of a Voronoi polygon, where the micelle depth at **r**_*j*_ is equal to the depth of the anchor point which subtends the smallest angle *𝜃*_*ij*_. This produces a discontinuous surface, reminiscent of that presented in [47]. Images of the intrinsic surface for small *λ* (*λ* = 0) and large *λ* (*λ* = 1000) are shown in Fig. 2. Clearly, the value of *λ* should be chosen to produce a surface for which the micelle depth is close to the value of |**s**_*i*_| in the vicinity of anchor point *i*, yet changes smoothly and continuously in regions between anchors. The value of *λ* can be systematically chosen by establishing the typical angle between the vectors {**s**_*i*_} of nearest-neighbor anchor points throughout the trajectory, $\bar {\theta }$, and then use this angle to determine *λ* for a specific system: $\lambda =1/\bar {\theta }^{2}$. In this way, the decay constant of the function is on the order of the typical separation of nearest-neighbor anchors. This method yielded the choice of *λ* = 15 for the system in the current study and was used for all analysis performed.

**Fig. 3:**
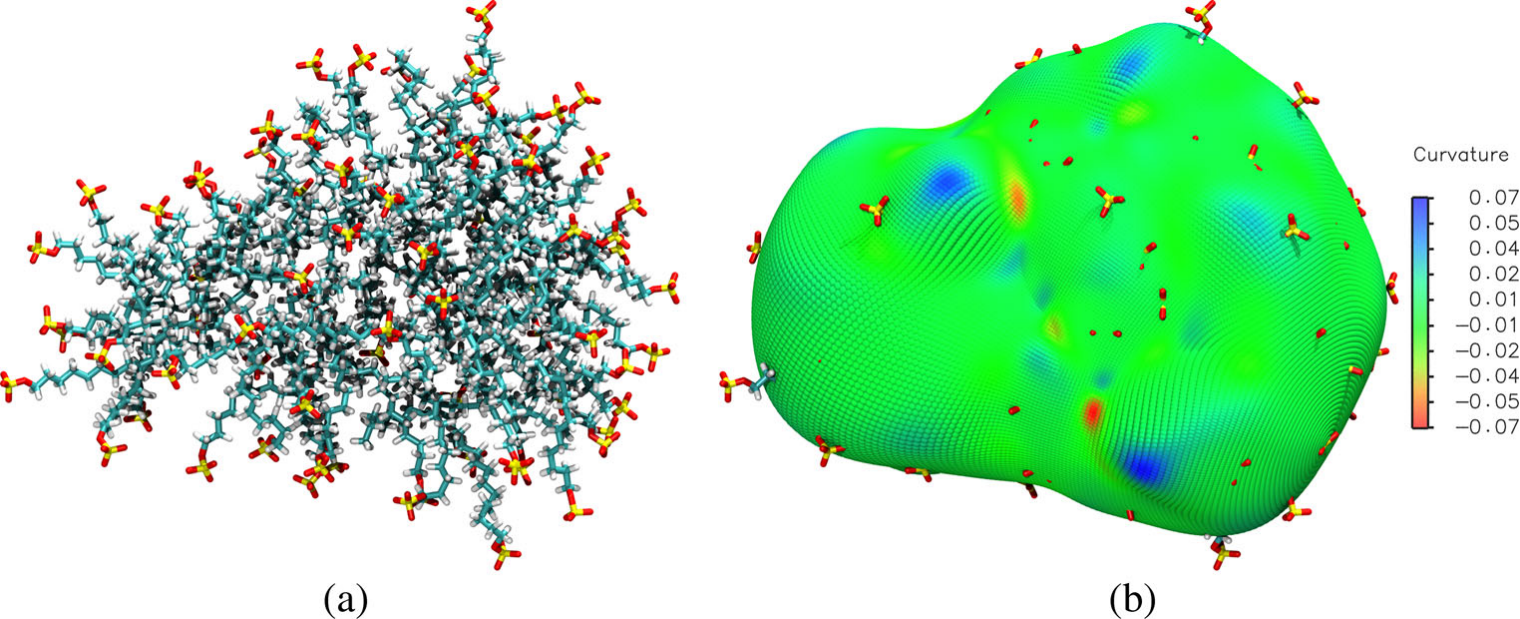
**a**) The final snapshot in the production simulation of the SDS+TP micelle. Atoms with the colors *cyan*, *grey*, *red*, and *yellow* are used to represent the elements: carbon, hydrogen, oxygen, and sulfur in SDS molecules, respectively. TP molecules are omitted from this image for clarity. **b**) The micelle intrinsic surface superimposed over the last configuration of the production simulation. The color of the surface represents the local Gaussian curvature, as defined on the *color scale bar on the right*

Another complicating phenomena when trying to identify the surface of soft interfaces is the protrusion of individual molecules further into the water phase, as is shown in Fig. 3a. By choosing *λ* as discussed above, we ensure that a protruding surfactant molecule does not especially influence the local definition of the micellar surface. This value of *λ* ensures that the micelle depth in the vicinity of a dislodged surfactant will be heavily influenced by the other anchor points, which will effectively drag the surface back towards the rest of the micelle, as illustrated on the right-hand side of Fig. 3b, as opposed to creating discontinuities in the surface like those in Fig. 2b. Figures 2a & b and 3b are produced by evaluating the intrinsic surface at discrete points across the full range of the polar angles, *𝜃* and *ϕ*, and building a representation of the intrinsic surface of the entire micelle.

### Radial density

Micelles are dynamic structures which, in the current case, adopt a quasi-spherical shape and are generally assembled such that the hydrophilic headgroups are in contact with water whilst the solvophobic tails are contained in the hydrophobic core of the micelle. Commonly in the literature, the structure of micelles studied using MD simulations is quantified by producing radial density plots of various different atomic species contained within the system. Such plots are constructed by first calculating **c**_m_, and next counting the number of atoms of a given species contained within spherical shells at varying distances, *d*_cm_, from **c**_m_. Finally, the average density is obtained by dividing the total count for each shell by both the total number of snapshots used in the analysis and the volume of each spherical shell.

### Intrinsic density

To determine the intrinsic density, the procedure is very similar to that for the radial density, except that the density is expressed in terms of *d*_int_ rather than *d*_cm_. One ramification of this variable change is the increased complexity of determining the volume of the spatial intervals used in the intrinsic density calculation. When measuring the radial density, the volume of a spatial interval centered at *r* is calculated by evaluating the integral over the spherical surface:

2 $$ V(r)={\int}^{r+dR/2}_{r-dR/2} {\int}^{2\pi}_{0} {\int}^{\pi}_{0} r^{{\prime}2} \sin{\theta} d\theta d\phi dr^{\prime}  $$

where *r* ^′^, *𝜃*, and *ϕ* are spherical polar coordinates and *d* *R* is the width of the spatial interval. From this, we obtain an analytic expression for the volume of a spatial interval centered at *r*: $V(r)=\frac {4}{3}\pi [(r+\frac {dr}{2})^{3}-(r-\frac {dr}{2})^{3}]$, where *r* denotes the distance between **c**_m_ and the center of the spatial interval. For the intrinsic density, the volume of the spatial intervals can in principal be calculated by evaluating a similar triple integral over the intrinsic surface as that presented in Eq. 2. However, in this instance, *r* ^′^ is a function of *𝜃* and *ϕ* (i.e., *r* ^′^ = *r* ^′^(*𝜃*,*ϕ*)), therefore this integral cannot be solved analytically and to solve it numerically for each spatial interval at every snapshot would be a laborious task. Instead, we estimate the volume as follows. First, the simulation box is divided into a 3-D grid with a specified grid width, *d*_g_ (which should be no larger than the width of the spatial interval). The volume of a spatial interval is then estimated by summing up the volume of each grid element, $d_{\mathrm {g}}^{3}$, which resides within the spatial interval. The average intrinsic density is obtained by dividing the number of atoms located within a spatial interval by the instantaneous volume of the spatial interval, and averaging over many snapshots taken from a stable part of the simulation trajectory.

### Water orientation

In the current study, we are investigating the encapsulation of poorly water soluble TP molecules within an SDS micelle. This process is affected not only by the properties of the micelle itself but also by the structure of the water molecules surrounding the micelle. An instructive way to study this is through orientation profiles of water molecules. We define the dipole vector of a water molecule, $\hat {\mathbf {p}}$, as a unit vector pointing from the Ow atom to the geometric center of the two Hw atoms in a water molecule. Then we compare the orientation of this vector with $\hat {\mathbf {r}_{j}}$, which is a unit vector pointing from **c**_m_ to Ow. The dot product of these vectors describes the orientation of a water molecule: cos(*𝜃*). When cos(*𝜃*) = 1.0, the water molecule is oriented such that its dipole vector is in perfect alignment with **r**_*j*_, when cos(*𝜃*) = −1.0, the water molecule is oriented such that its dipole vector forms a 180 ^∘^ angle with **r**_*j*_. Probability density distributions can be constructed that describe the likelihood of observing a water molecule with a particular orientation at a given value of the distance:

3 $$ \rho_{d}(\cos{(\theta)}) = \left\langle \frac{1}{N_{d}} \sum\limits_{j=1}^{N_{\text{wat}}} \delta(d-d_{j}) \delta(\cos{(\theta)}-\cos{(\theta_{j} )})\right\rangle  $$

where *ρ*_*d*_(cos(*𝜃*)) is the probability density of finding a water molecule with an orientation, $\cos {(\theta )}=\hat {p} \cdot \hat {r_{j}}$, for a given value of distance *d*, *N*_*d*_ is the instantaneous number of water molecules located at *d*, the summation indexed by *j* runs over all *N*_wat_ water molecules in the system, and *δ* denotes the Dirac delta function.

The radial water orientation profile is obtained by calculating the function defined in Eq. 3 using *d*_cm_ as the distance and thus describes the orientation of water molecules as a function of their distance away from **c**_m_. Alternatively, the intrinsic water orientation profile is produced by calculating the function defined in Eq. 3 using *d*_int_ as the distance, which describes the orientation of water molecules as a function of their distance away from the micelle intrinsic surface.

### Gaussian curvature

In order to characterize the curvature of the micelle, the coordinates of the molecules in the micelle first need to be translated such that the **c**_m_ is positioned at the origin. Then the micelle’s intrinsic surface can be expressed by a vector in Cartesian coordinates, **r**, as a function of the azimuthal and zenith angles *𝜃* and *ϕ*, respectively. From this formulation, the local Gaussian curvature can be calculated as the ratio of the first and second fundamental forms of the intrinsic surface function, *r*(*𝜃*,*ϕ*):

4 $$ \kappa = \frac{\det{(\mathbf{II})}}{\det{(\mathbf{I})}} = \frac{\text{LN}-\text{M}^{2}}{\text{EG}-\text{F}^{2}} $$

where *κ* is the Gaussian curvature, *E*, *F*, and *G* are coefficients of the first fundamental form, and *L*, *N*, and *M* are coefficients of the second fundamental form [62] (we refer the reader to the S.I for a more detailed explanation of the Gaussian curvature). This provides a description of the local curvature of the micelle intrinsic surface, which is a useful property that has yet to be incorporated into the analysis of micelles in the literature. Positive values of curvature correspond to either convex or concave regions of the surface, whereas negative curvature values correspond to saddle points. As an illustration of how the curvature can be incorporated into analysis of micellar systems, we investigate the lifetimes of highly curved micelle regions in the hope of improving our understanding of the typical timescales over which the micelle geometry fluctuates. This is achieved by choosing a curvature magnitude threshold and then producing histograms of the time taken for the curvature of a point on the micelle surface to change in the two following ways: (i) to decrease from the positive threshold value to 0, corresponding to a convex/concave region flattening out and (ii) to increase from the negative threshold value to 0, corresponding to a saddle point flattening out.

## Results

In this section, we present the results of the analysis outlined in the previous section. This analysis has allowed us to study the structure of the resulting micelle, the encapsulation of TP within the micelle, and the orientation of water molecules around the micelle. In particular, where reasonable, we will highlight the differences in the results when describing the micellar interface using the intrinsic surface.

### Density plots

**Fig. 4:**
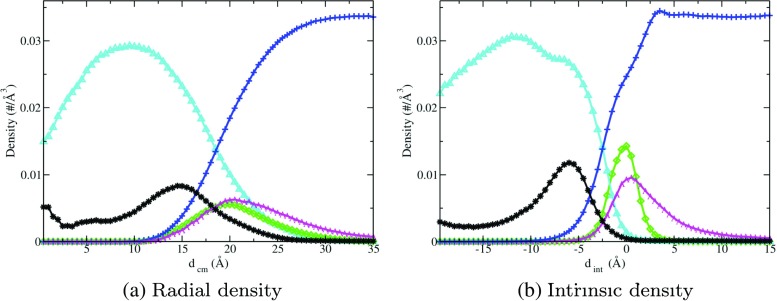
The **a**) radial and **b**) intrinsic density profiles, respectively, for the SDS+TP micelle system, in which the colors *green*, *cyan*, and *magenta* are used to depict the density of oxygen, carbon, and sodium atoms in SDS, respectively. *Blue* and *black* are used to show the density of oxygen atoms in water molecules and carbon atoms in TP, respectively. Note that the density of Na ^+^ has been multiplied by ten to improve clarity

Figure 4a and b show radial and intrinsic density profiles respectively for the various different atomic species in the SDS+TP system. The intrinsic density plots are shown with the range − 20.0 ≤ *d*_int_ ≤ 15.0 such that a meaningful comparison can be drawn between these and the radial density plots that have the same length scale (0.0 ≤ *d*_cm_ ≤ 35.0).

In general, the oxygen atoms in SDS molecules, O _DS_, provide a good indication of the position of the surfactant headgroups. One can estimate the average micelle radius from the radial density plots by taking the value of *d*_cm_ corresponding to the peak density of the O _DS_ atoms. The O _DS_ radial density profile (green curve in Fig. 4a) shows a broad distribution around the peak, which is situated at 22.5 Å providing an estimate of the micelle radius. The smearing of the distribution arises due to micellar shape fluctuations and highlights the necessity of incorporating a rigorous micelle surface definition. Conversely, the O _DS_ intrinsic density profile (green curve in Fig. 4b) is relatively sharp, showing a strong localization of O _DS_ around the interface. This is an unsurprising result considering that the O _DS_ atoms are all bonded to sulfur atoms, which are used as the anchors to construct the micelle/water surface.

The radial and intrinsic density profiles of oxygen atoms in water molecules, Ow, are shown by the blue curves in Fig. 4a and b, respectively. At sufficiently large distance values, the number density of Ow atoms converges to 0.033 Å ^−3^ corresponding to a density of 1 g/ml, which is consistent with the known bulk density of water. The Ow radial density decreases monotonically to zero as *d*_cm_ → 0. The intrinsic density profile of Ow atoms (blue curve in Fig. 4b) differs somewhat from the radial density in that it exhibits a small interfacial peak located at *d*_int_ = 3.5 Å. This peak corresponds to the attraction between the polar water molecules and the highly charged micelle surface. This is a subtle yet important structural feature which is undetected by the radial density plot. In both density plots, as the distance values decrease corresponding to positions in the micelle interior, the density of Ow atoms decays significantly but is non-zero in the entire range. To quantify this effect, the intrinsic density of Ow decreases by 77% from *d*_int_=2.5 Å to *d*_int_=-3.5 Å. This provides an accurate estimate of the depth of penetration of water into the micelle interior as we have used a rigorous definition of the micelle intrinsic surface. From this, it can be deduced that a small amount of water is able to penetrate to the center of the micelle. The radial density plots generally show that the SDS monomers are arranged such that the headgroups are in contact with the water, whereas the tails are contained in the micellar core forming a hydrophobic environment. The intrinsic density plots suggest the same basic micelle shape and properties, however, they also contain more detail about the structure of atomic species at the interface including the interfacial water peak.

The cyan curves in Fig. 4a and b represent the radial and intrinsic density profiles, respectively, of carbon atoms in SDS, C _DS_. At large distance values corresponding to the bulk water region, the C _DS_ density obtained from both methods is equal to zero, indicating that the carbon chains are not located in the bulk water. As the distance value decreases, an increase in the C _DS_ density is observed from both methods until peaks in the density are reached. These peaks occur at the approximate center of the surfactant chains (*d*_cm_ = 10.5 Å and *d*_int_=-11 Å). As *d*_cm_ → 0, the C _DS_ radial density also tends to 0. Conversely, as *d*_int_ decreases past the peak, the C _DS_ density decreases monotonically but does not reach 0 for the range shown. This is because there is typically a non-negligible volume associated with points at these small values of *d*_int_ in which C _DS_ are found, as opposed to the vanishingly small volume associated with *d*_cm_ ∼ 0 in the radial density plot.

The density profiles for the Na ^+^ counterions (magenta curves in Fig. 4a and b) are non-zero at large distances, indicating that Na ^+^ counterions occupy the bulk water region. Then, as the distance values decrease, the Na ^+^ density increases approaching the interface where both methods predict peaks indicating localization of counterions at the interface. Inspection of the Na ^+^ intrinsic density reveals a more prevalent density peak, situated at *d*_int_ = 1 Å, which is almost twice the value of the corresponding radial density peak located at *d*_cm_ = 21.5 Å. The extent of the localization is once again realized through the intrinsic density plot, in an analogous way as for the O _DS_ atoms as described above. Additionally, another discrepancy between the two density calculations is observed. The radial density profiles for Na ^+^ and O _DS_ atoms are alike in that the distribution of their densities as a function of *d*_cm_ are similar in both shape and peak position. This suggests that these atomic species exhibit similar behavior in regard to their relative occupancy of different spatial regions of the micelle. It becomes clear, however, from the intrinsic profiles that the true density distribution of Na ^+^ is much broader compared to that of O _DS_, extending to large values of *d*_int_ whereas the O _DS_ intrinsic density reveals a much more pronounced density peak than in the radial profile, which is indicative of a strong affinity with the micelle interface.

The position of TP molecules was studied by calculating the density of the carbon atoms within them, as shown by the black curves in Figs. 4a and b. Considering just the carbon atoms, C _TP_ provides a good indication of the TP’s whereabouts given that the molecular weight of a TP molecule consists predominantly of carbon. The density of C _TP_ vanishes at large distances in both density calculations and thus we deduce that TP is not found at large distances away from the micelle in the bulk water. Then, as the distance decreases, the C _TP_ density begins to increase gradually until it peaks within the micelle interior. Although the radial and intrinsic C _TP_ density profiles both predict density peaks within the micelle, located at *d*_cm_ = 15.5 Å and *d*_int_ = −6 Å respectively, the two density curves are significantly different in the description of the region where the C _TP_ density becomes non-zero. The radial profile suggests that there is an appreciable C _TP_ density within the water surrounding the micelle. On the contrary, the intrinsic density profile shows that the density is effectively 0 at *d*_int_ = 0, which shows that the TP molecules are situated at the micelle’s interfacial region and not in the bulk water as the radial density plot suggests. In summary, the radial density plot suggests that the TP molecules are situated both at the interfacial region and within the palisade layer of the micelle, whereas the intrinsic density profile suggests that the TP molecules are situated only within the palisade region.

### Water orientation

The structure of interfacial water molecules is highly relevant to the study of encapsulation properties of micelles. Solutes must overcome an energy barrier which arises from the highly ordered water structure in the vicinity of the micelle. Studying the structure of water surrounding the micelle could provide some indications as to the micelle’s potential as a solubilizing agent.

**Fig. 5:**
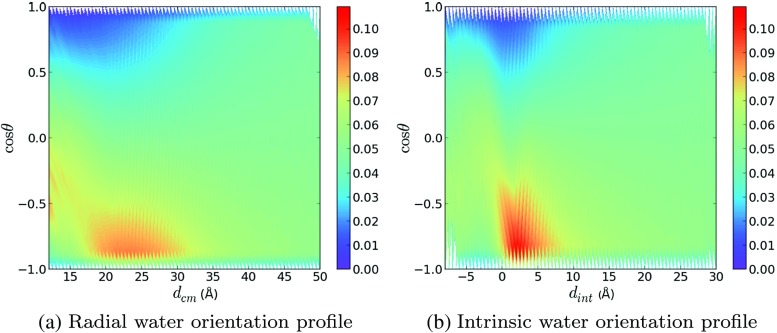
Probability density plots for **a** the radial orientation and **b**) the intrinsic orientation of water molecules

The orientation of water molecules was investigated by studying both the radial and the intrinsic water orientation profiles, as outlined earlier in “Water orientation”. The radial and intrinsic water orientation profiles are shown in Fig. 5a and b, respectively. In both of these plots, the *x*-axis represents the distance (*d*_cm_ or *d*_int_) and the *y*-axis represents cos(*𝜃*), where *𝜃* is the water dipole angle with respect to the vector pointing from **c**_m_ to the Ow atom. Note that the integral of the probability density over cos(*𝜃*) for a fixed value of the distance variable is normalized to 1.

The radial water orientation profile, Fig. 5a, shows that for large distances away from the micelle, water molecules have no orientational preference when averaged over many snapshots. This is because the interaction with the charged micelle interface is very weak at large distances. This explains the approximately flat distributions of cos*𝜃* at values of 35 ≤ *d*_cm_ ≤ 50. As the value of *d*_cm_ decreases, the orientation of the water molecules is significantly affected by the interaction with the electric field arising from the micelle surface. This is evident from the yellow tinge at cos*𝜃* ∼−0.9, *d*_cm_ ∼ 35 Å. This corresponds physically to an increased tendency of water molecules to orient their dipoles such that the Hw atoms are closer to the micelle surface than the Ow atoms. This effect becomes more prominent as *d*_cm_ decreases further as is clear from the broad red region in the vicinity of the micelle interface: 18 ≤ *d*_cm_ ≤ 30. As *d*_cm_ decreases further still, the distribution shifts back towards larger values of cos(*𝜃*) as seemingly water molecules that have penetrated through the micelle interface attempt to reorient to allow favorable interactions between Hw atoms and surfactant headgroups. It seems from Fig. 5a that this reorientation affects water molecules as close as 8 Å away from **c**_m_, shown by the yellow/green tinge on the far left of the plot, this would correspond to molecules which are ∼ 14 Å deep in the micelle core.

Inspection of the intrinsic water orientation profile reveals qualitatively similar behavior to that arising from the radial water orientation profile. At large values of *d*_int_, corresponding to large distances away from the micelle surface, the distribution of water molecule orientations is flat, as we would expect. Whilst the intrinsic water orientation profile also predicts the reorientation of water molecules in the vicinity of the micelle interface, it occurs over a much smaller distance range: 0 ≤ *d*_int_ ≤ 5 Å. This region exhibits a comparatively sharp probability density peak, shown by the bright red region in Fig. 5b, located at cos*𝜃* = −0.9, *d*_int_ = 2.5 Å. The location of this peak is in direct correspondence with the location of the interfacial water peak in the intrinsic density profile as we would expect. The smaller distance range in which the reorientation occurs results in a V-shape trend where the probability density is severely skewed towards smaller values of cos*𝜃* and then skewed back in the opposite direction. Interestingly, the intrinsic water orientation profile shows that the effect of the interface on the reorientation of water molecules is highly localized to those in the vicinity of the interface. At *d*_int_ ≤ -5 Å, the water orientation probability density distribution is more or less flat like in the bulk water. It seems then that the radial water orientation profile, in this instance at least, overestimates the range of interaction in which the orientation of water molecules is affected by the charged micelle surface.

### Surface curvature lifetimes

Prior to this study, local Gaussian curvature had yet to be incorporated into analysis of micelles in the literature. Here, we attempt to utilize this as an additional way of characterizing micelle shape and dynamics. A curvature tolerance of 0.05 Å ^−1^ was used for the SDS+TP micelle system, as this encompassed regions of the surface that were highly curved, yet contained a sufficient number of examples for adequate statistics to be collected.

**Fig. 6:**
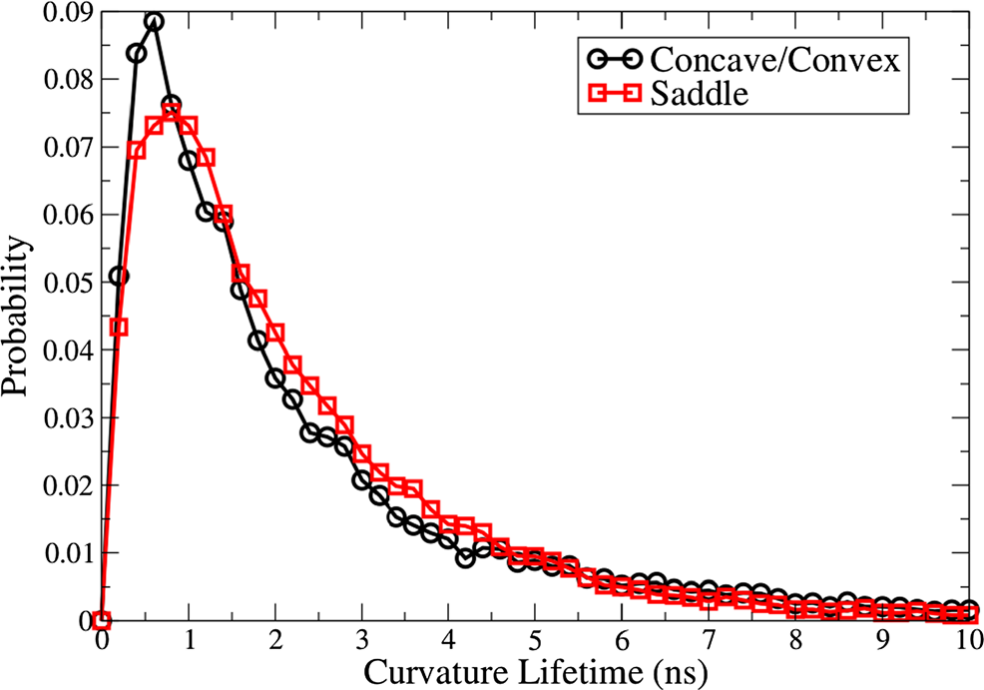
Probability histograms of curvature lifetimes in units of nanoseconds for both convex/concave and saddle points

The curvature lifetime can be thought of as the time taken for a surface point, which has an absolute value of curvature equal or greater than the tolerance, to decrease to 0. Histograms have been produced (Fig. 6) that show the probability of different curvature lifetimes for concave/convex points (black curve) and saddle points (red curve) throughout the production simulation. As curvature lifetime increases, the curves in Fig. 6 increase sharply from 0 to peaks in probability at values of 0.6 ns and 0.8 ns for concave/convex and saddle regions, respectively. As the lifetime increases further, the curves decrease exponentially and tend towards 0 at large values. The probability of a highly curved surface region existing for longer than 8 ns is practically negligible (2.5 × 10 ^−3^).

In general, the typical lifetime of a region of high curvature is fairly small at less than 10 ns, however saddle points appear to be more stable than concave/convex regions with a slightly larger peak lifetime and a higher probability of moderate lifetimes as shown by the red curve exhibiting larger probability values within the range ∼ 2.0–4.5 ns. While further investigation of this observation is necessary, one reason why concave/convex points are less stable than saddle points is that concave/convex portions of the micelle are often a result of a protruding surfactant molecule from the local interface and therefore potentially exposing some less favorable interaction sites to the aqueous environment, whereas the saddle points are usually formed by numerous surfactant molecules at each distance from the surface of the micelle, which can stabilize each other through various interaction mechanisms including salt bridges. For lifetimes > 5 ns, the two curves coincide almost exactly.

## Conclusions

In this study, we have investigated the structural and interfacial properties of an SDS micelle in the presence of TP molecules using all-atom MD simulations. In doing so, we have proposed a novel method of constructing the continuous micelle intrinsic surface that is calculated from a finite number of anchor points, specifically the sulfur atoms in the surfactant headgroups. We argue in particular that when studying the structure of micelles, the more informative variable to consider is the distance to the micelle surface, as opposed to the distance to the micelle centre of mass, as is routinely used in the field at the present time. Our reasoning for this is that the former provides a more accurate definition of the micelle/water interface, whereas the latter is indiscriminate to capillary wave fluctuations, which result in a smeared representation of the micelle/water interface.

The overall structure and the hydrophobic core of the micelle were each studied using both radial and intrinsic density profiles. In addition to these, the water orientation profiles were reported to study the structure of the water molecules surrounding the micelle, again constructed separately as radial and intrinsic functions. In both of these analyses, use of the intrinsic surface resulted in a more detailed and insightful description of the micelle properties. In particular, the intrinsic density profile predicts an interfacial water peak whereas the radial density profile is unable to detect this subtle yet important structural feature. The position of the TP molecules in relation to the micelle was inconclusive from the radial density profile because of the broad range of the density distribution of C _TP_ atoms, which spanned from the micelle core into the bulk water region. The intrinsic density profile, however, shows that the density of C _TP_ atoms decays to 0 at the micelle surface, and thus we conclude that TP resides in the micelle core region.

The orientation of water molecules was studied by calculating radial and intrinsic orientation profiles. The intrinsic water orientation profile reveals that water molecules in the vicinity of the micelle surface have a particularly strong tendency to orient such that their dipole vector is pointing towards the surfactant headgroups. The radial water orientation profile also predicts this reorientation of water molecules, however the detail about where it occurs is once again smeared due to the lack of an accurate interface definition.

The local Gaussian curvature of the intrinsic surface was examined to investigate the micelle shape dynamics from a new perspective. This analysis reveals that fluctuations in the micelle shape occur on the order of ns, with saddle point regions being slightly more robust than concave/convex points as indicated by the longer expected curvature lifetime.

Throughout this study, incorporating the intrinsic surface into analysis of the SDS+TP micelle has led to a more insightful analysis over the radial counterparts in both the density plots and the water orientation profiles. Moreover, constructing the intrinsic surface and collating curvature measurements has uncovered a new perspective on the micelle shape dynamics, which has been very instructive. In this manuscript, we have explored a limited number of applications of intrinsic surface analysis, however this can be utilized even further to study many more interesting properties of micelles and nanoparticles in general. One example of such a property is the depth of insertion of TP molecules into the micelle. This is crucial to our understanding of the encapsulation process of drugs within micelles, which in turn affects the ability of SDS micelles to act as vectors in oral drug delivery. In the future, we plan to build upon our previous work [17, 41] by performing well-tempered metadynamics simulations to obtain the free energy landscape as a function of depth of insertion of various different testosterone derivatives into surfactant micelles. The collective variables used in metadynamics calculations must be continuous functions to ensure energy conservation. This provides further justification for our proposed method, as the discontinuous surface yielded from the work of Chowdhary et al. [47] is insufficient for such use. By constructing the free-energy landscape of the insertion of solutes into micelles, we strive to gain a greater understanding of these molecular systems and how the chemistry of the surfactant and drug molecule affects the encapsulation process.
